# Aggregation of the Protein TRIOBP-1 and Its Potential Relevance to Schizophrenia

**DOI:** 10.1371/journal.pone.0111196

**Published:** 2014-10-21

**Authors:** Nicholas J. Bradshaw, Verian Bader, Ingrid Prikulis, Angelika Lueking, Stefan Müllner, Carsten Korth

**Affiliations:** 1 Department of Neuropathology, Heinrich Heine University, Düsseldorf, Germany; 2 Protagen AG, Dortmund, Germany; Universitat Autònoma de Barcelona, Spain

## Abstract

We have previously proposed that specific proteins may form insoluble aggregates as a response to an illness-specific proteostatic dysbalance in a subset of brains from individuals with mental illness, as is the case for other chronic brain conditions. So far, established risk factors DISC1 and dysbindin were seen to specifically aggregate in a subset of such patients, as was a novel schizophrenia-related protein, CRMP1, identified through a condition-specific epitope discovery approach. In this process, antibodies are raised against the pooled insoluble protein fractions (aggregomes) of post mortem brain samples from schizophrenia patients, followed by epitope identification and confirmation using additional techniques. Pursuing this epitope discovery paradigm further, we reveal TRIO binding protein (TRIOBP) to be a major substrate of a monoclonal antibody with a high specificity to brain aggregomes from patients with chronic mental illness. *TRIOBP* is a gene previously associated with deafness which encodes for several distinct protein species, each involved in actin cytoskeletal dynamics. The 3′ splice variant TRIOBP-1 is found to be the antibody substrate and has a high aggregation propensity when over-expressed in neuroblastoma cells, while the major 5′ splice variant, TRIOBP-4, does not. Endogenous TRIOBP-1 can also spontaneously aggregate, doing so to a greater extent in cell cultures which are post-mitotic, consistent with aggregated TRIOBP-1 being able to accumulate in the differentiated neurons of the brain. Finally, upon expression in Neuroscreen-1 cells, aggregated TRIOBP-1 affects cell morphology, indicating that TRIOBP-1 aggregates may directly affect cell development, as opposed to simply being a by-product of other processes involved in major mental illness. While further experiments in clinical samples are required to clarify their relevance to chronic mental illness in the general population, TRIOBP-1 aggregates are thus implicated for the first time as a biological element of the neuropathology of a subset of chronic mental illness.

## Introduction

Schizophrenia, along with the related conditions bipolar disorder and major depression, are devastating and often chronic conditions with a strong genetic basis that has only partially been explained to date by means of conventional and genome-wide genetic association and linkage studies [1]. In Alzheimer's disease, by comparison, significantly more progress in understanding the condition's pathological mechanism has arisen through the identification of mechanisms of assembly of the Aβ peptides into plaques [2], characteristic of the disease, than through traditional genetic approaches [3]. While no such large plaques or aggregated protein structures exist for major mental illnesses such as schizophrenia, we have previously put forward the hypothesis that the formation of micro-aggregates or assemblies of specific proteins within the neurons and/or other cells of the brain may be hallmark of such psychiatric illnesses and account for the chronic nature of these conditions in some patients [4]. Initially, we focussed on proteins encoded for by known mental illness risk genes, and by this approach identified both Disrupted-In-Schizophrenia 1 (DISC1) and dysbindin as showing aberrant aggregation in a subset of patients with schizophrenia, bipolar disorder or major recurrent depression [5], [6]. In a hypothesis-free approach, we further identified collapsin response meditator protein 1 (CRMP1, also known as DPYSL1) as the epitope for an antibody which could discriminate between a pool of aggregated proteins (aggregomes) derived from brain samples of patients with schizophrenia compared to an equivalent pool from control individuals [7]. In a related, proteomic approach we used the identification of a proteostatic signature represented by the accumulation of specific insoluble proteins to identify molecular circuitry associated with failure in cognitive features [8].

In this study we further develop our epitope discovery paradigm, revealing TRIO binding protein (TRIOBP) to be the major substrate of a monoclonal antibody with high specificity to schizophrenia brain aggregomes. The *TRIOBP* gene encodes for multiple splice variants including *TRIOBP-1* which lies at the 3′ end of the locus and *TRIOBP-4* which lies at the 5′ end of the locus, sharing no exons with *TRIOBP-1* [9], [10]. Additionally long isoforms of more than 200 kDa exist such as *TRIOBP-5* which incorporates both the *TRIOBP-1* and *TRIOBP-4* exons [10]. Both the protein encoded for by the major 3′ isoform TRIOBP-1, also known as Tara, and the major 5′ isoform of mouse, TRIOBP-4, have been shown to be vital for actin polymerisation [9], [11], with knockdown of TRIOBP by siRNA being useable as a tool to block F-actin formation in the cell [12]–[14]. Of these variants, the 3′ variant TRIOBP-1 is the most ubiquitously expressed, while the 5′ variants are mainly found in the retina and inner ear [10]. Mutations in these latter variants are associated with deafness [10], [15]–[17] due to defects in stereocilia function in the inner ear [18].

While the *TRIOBP* gene has not previously been implicated in chronic mental illness directly, its expression was found to be altered by a haplotype of the *NDE1* gene [19] which has itself been found to be associated with schizophrenia [20] and encodes for a protein known to be of significant importance for cortical neurodevelopment (reviewed [21]).

Here, we report on thorough biochemical and cell biological analyses of the aggregation propensity of TRIOBP, identifying the TRIOBP-1 splice variant as the principal aggregation-prone species, yielding insight into the mechanisms by which this arises and demonstrating that it has the capability to alter the morphology of neuron-like cells in culture.

## Materials and Methods

### Antibodies and DNA constructs

Antibody 6H11 has been described previously [7], but briefly was raised against the pooled insoluble fractions of 15 post mortem brain samples from patients with schizophrenia supplied by the Stanley Medical Research Institute and screened against pooled corresponding fractions from non-schizophrenia brains. Commercial antibodies were used against GAPDH (Santa Cruz, sc-137179), TRIOBP-1/5 (Sigma, HPA003747) and β3-tubulin (TUJ1 clone, Covance, MMS-435P). AlexaFluor 594 Phalloidin was purchased from Life Technologies.

Constructs encoding CRMP1 variants have been described previously [7]. Constructs encoding GFP fused to TRIOBP-1, 4 and 5 [18] were gifts from Dr. Thomas Friedman (National Institutes of Health, Rockville, MD, USA). To delete the Pleckstrin homology domain of the construct encoding TRIOBP-1, EcoRI restriction digest sites were introduced using the QuikChange Lightning Multi-Site-Directed Mutagenesis kit (Agilent) and the primers cgggtgccgccatgaattccgatctgctcaacttcaag and tggattgaggctctcaggaattctgtgcggccaacttcagc. The construct was then EcoRI-digested, the two fragments separated on an agarose gel and the major fragment religated using T4 DNA Ligase (New England BioLabs). All constructs were confirmed by sequencing.

For recombinant expression of human TRIOBP-1, the open reading frame was codon optimised for *E. coli* expression and synthesised with flanking attB sites by GeneArt (Life Technologies) and then subsequently transferred by recombination into the pDONR/Zeo (Life Technologies) and pETG-40A (A. Geerlof, a gift from the Protein Expression and Purification Core Facility at EMBL, Heidelberg, Germany) vectors using BP and LR clonase enzymes (Life Technologies). A control MBP construct was generated by similarly transferring the empty recombination cassette of the pENTR1A no ccDB vector [22] (Addgene, clone 17398) into pETG-40A. All constructs were confirmed by sequencing.

### Cell culture

The NLF (Children's Hospital of Philadelphia, Philadelphia, USA) and NMB [23] neuroblastoma cell lines cells were grown in RPMI 1640 containing 10% foetal calf serum, 2 mM L-glutamine, penicillin and streptomycin. The SH-SY5Y [24] neuroblastoma cell line was grown in D-MEM/F-12 containing 10% foetal calf serum, 1x MEM non-essential amino acid solution, penicillin and streptomycin. The Neuroscreen-1 cell line (Thermo Scientific) was cultured in RPMI 1640 media containing 10% horse serum, 5% foetal calf serum, 2 mM L-glutamine, penicillin and streptomycin. The CL4 epithelial cell line [25] was a gift from Dr. James Bartles (Northwestern University, Chicago, IL, USA) and was cultured in MEMα medium without ribonucleotides or deoxyribonucleoides, containing 10% foetal calf serum, penicillin and streptomycin. All cell media was from Life Technologies.

For one experiment, SH-SY5Y cells were differentiated by treatment for 3 days with 10 µM retinoic acid, followed by 3 days treatment with 80 nM phorbol-12-myristate-13-acetate (PMA), based on published protocols [26]. Similarly, NMB cells were differentiated by treatment for 6 days with 20 µM dopamine, replacing media and additives after 3 days [27].

Primary neurons were prepared from the cortices of E18 Sprague-Dawley rat embryos, seeded on poly-L-ornithine coated plates and cultured in neural basal medium containing B27 supplement, GlutaMAX, penicillin and streptomycin (all from Life Technologies). Depending on the experiment, neurons were cultured for 14–21 day *in vitro* before analysis.

For experiments requiring the over-expression of TRIOBP proteins, DNA constructs were transfected into cell lines using Metafectene (Biontex Laboratories) or into primary neurons using Lipofectamine 2000 (Life Technologies) for a period of 16 hours in antibiotic-free media, according to manufacturers' instructions. Cells and neurons were then lysed 8 hours later, for a total of 24 hours after the initiation of transfection.

### Antibody epitope identification

Two separate preparations of antibody 6H11 were screened on UNIchip AV-400 protein microarray slides under GLP conditions (Protagen AG) as described previously [28]. Briefly, these chips contain random combinations of human recombinant proteins in quadruplicate, against which antibodies are screened to detect potential antigens. Chips were incubated with the antibody of interest followed by secondary antibody and then read on a ScanArray 4000 confocal microarray reader (Perkin Elmer Life Science), before data analysis using GenePix Pro 6.0 software (Molecular Devices). For confirmation, chips containing selected antigens at a range of decreasing concentrations from 20 to 0.002 fmol were employed.

### Identification of putative aggregation regions and secondary structure through bioinformatics

Amino acid sequences of *TRIOBP* splice variants were analysed using four aggregation/amyloid propensity prediction servers, which in total employ six different measures of predicted aggregation. These were as follows: AGGRESCAN [29], bioinf.uab.es/aggrescan, taking an A4V value of 0 as the threshold for aggregation for each individual residue; FoldAmyloid [30], bioinfo.protres.ru/fold-amyloid/oga.cgi, using default parameters, threshold  = 21.4; ProA [31], www.abl.ku.edu/ProA, using both the ProA-SVM and ProA-RF propensity statistics, threshold 0 0.5; Tango [32]–[34], tango.crg.es, both alpha helix and beta sheet aggregation using default parameters, threshold  = 5%. Putative aggregated regions were defined as stretches of 5 or more consecutive amino acids which were each predicted to have aggregation propensity by at least 3 of these 6 methods based on the thresholds listed above. Secondary structure of the proteins was predicted using PSIPRED [35], bioinf.cs.ucl.ac.uk/psipred, and COILS [36], www.ch.embnet.org/software/COILS_form.html. Disordered regions were investigated using the metaPrDOS meta server [37], prdos.hgc.jp/cgi-bin/meta/top.cgi. Potential functional features were identified using ScanSite with stringency set to “high” [38], scansite.mit.edu/motifscan_seq.phtml.

### Western blotting

Western blotting was performed according to standard protocols. Following transfer, protein on nitrocellulose membranes was detected using the primary antibodies listed above and visualised using either peroxidase-conjugated secondary antibodies (Thermo Scientific) and ECL Western Blotting Substrate (Thermo Scientific), or using IRDye secondary antibodies (LI-COR) and an Odyssey Clx infrared imaging system (LI-COR). For direct staining of total protein on a gel, InstantBlue Protein Stain (Expedeon) was used.

### Immunocytochemistry

Cells growing on glass coverslips were fixed for 10 minutes with 4% paraformaldehyde and permeabolised with 0.5% Triton X-100. Coverslips were blocked for 30 minutes with PBS/10% goat serum and then stained with primary (2 hours, diluted in PBS/0.03% BSA) followed by secondary antibodies (1 hour, diluted in PBS/10% goat serum), with three PBS/0.03% BSA washes between each step. Fluorescent phalloidin staining was performed for 20 minutes according to manufacturer's instructions. Coverslips were fixed with ProLong Gold mounting medium, containing DAPI (Life Technologies) and viewed on a Zeiss LSM-510 confocal microscope. Direct staining with florescent phalloidin was performed according to manufacturer's instructions.

### Insoluble aggregome purification

In order to purify the insoluble aggregomes of neuroblastoma cells, an adapted version of our previously published protocol [39] was used: Cells were grown to 90% confluency and then lysed with 50mM HEPES pH 7.5/300 mM NaCl/250 mM Sucrose/20 mM NaCl/1% NP-40/0.2% Sarcosyl containing Complete protease inhibitors (Roche) and DNaseI (Roche) and incubated at 37°C for 30 minutes. Protein concentrations of all samples involved in the experiment were then determined and normalised. A sample of the lysate was retained as a loading control and the remainder centrifuged at 1800×g for 30 minutes at 4°C. The supernatant was then removed, the pellet resuspended in 50 mM HEPES pH 7.5/1.5 M NaCl/250 mM Sucrose/5 mM EDTA/1% NP-40/0.2% Sarcosyl and centrifuged at 1800×g for a further 30 minutes at 4°C. Supernatant was removed, and this second pellet was resuspended in 50 mM HEPES pH 7.5/250 mM Sucrose/1% NP-40 and centrifuged at 1800×g for 30 minutes at 4°C. Supernatant was discarded and this third pellet was resuspended in pellet resuspended in 50 mM HEPES pH 7.5/0.2% Sarcosyl and centrifuged at 45,000×g for 30 minutes at 4°C. This final pellet was then resuspended in 1x loading buffer for running on an acrylamide gel.

### Recombinant protein production

MBP-tagged TRIOBP1 or MBP alone were expressed in BL21star cells. Cells were grown in LB media and induced with 1 mM IPTG for 3 hours at 37°C. Cells were spun down and suspended in 200 mM Tris pH 8, 200 mM NaCl, 1 mM DTT, 10% Glycerol, 20 mM MgCl_2_, 1.25 units/ml DNaseI, 1 mg/ml lysozyme and Complete protease inhibitor cocktail (Roche). Lysis was initiated with 1% Triton X-100 and lysate incubated at 4°C for 20 minutes. Cell debris was spun down and the supernatant was mixed in a 50∶1 ratio with Amylose Resin (New England BioLabs) and incubated overnight at 4°C. This slurry was then loaded into a column and washed with 20 column volumes of 5 mM Tris pH 8/50 mM NaCl/200 µM CaCl_2_. Protein was eluted by the addition of this same buffer containing 10 mM maltose.

### Neurite outgrowth assay

NS-1 cells were seeded sparsely on glass coverslips in RPMI 1640 media containing 10% horse serum, 5% foetal calf serum, 2 mM L-glutamine, penicillin and streptomycin. After 24 hours, cells were transfected with 0.5 µg per well of constructs encoding GFP, GFP-TRIOBP-1 or GFP-TRIOBP-4 using Lipofectamine 2000 (Life Technologies) according to manufacturer's instructions, for a total of 8 cover slips per DNA construct. Cells were then induced to grow for 96 hours with 50 ng/ml nerve growth factor (Life Technologies) in D-MEM media containing 1% foetal calf serum, penicillin and streptomycin. Media and growth factor were replenished after 48 hours. Cells were then fixed, blocked and stained with the TUJ1 antibody as described above. Coverslips were examined under a microscope and images taken of all cells showing fluorescence at 488 nm for which their neurites, as visualised by TUJ1 and its associated secondary antibody at 596 nm, could be clearly distinguished from surrounding cells. The first twenty such cells, or as many as could be found, were photographed per coverslip. From these images, dimensions of the cell body and Sholl analysis of the neurites were taken based on the TUJ1 staining using ImageJ software (http://imagej.nih.gov/ij) and the Concentric Circles plugin (http://imagej.nih.gov/ij/plugins/concentric-circles.html) as described previously [40]. Researcher performing the experiment was blinded as to the transfection status of each coverslip. At individual neurite lengths, the effect of transfected protein type generally was analysed using Kruskal-Wallis one-way analysis of variance, while the effect of GFP-TRIOBP-1 or GFP-TRIOBP-4 specifically versus GFP alone was analysed using the Mann-Whitney U test with Bonferroni correction for multiple testing. All other measures were analysed using two-tailed Welch's t-test with Bonferroni correction for multiple testing.

### Ethics statement

Human brain samples were obtained from the established Neuropathology Consortium cohort of the Stanley Brain Collection [41] of the Stanley Medical Research Institute (www.stanleyresearch.org). Use of these samples for this study was approved by the Ethics Commission of the Medical Faculty of the Heinrich Heine University, Düsseldorf. Use of the cortices of embryonic rats (embryonic day 18, Sprague-Dawley) to generate primary neurons has been granted by the Landesamt für Natur, Umwelt und Verbraucherschutz (State Agency for Nature, Environment and Consumer Protection), North Rhine-Westphalia, Germany and was performed in accordance with European and national animal care regulations.

## Results

### Protein aggregation of TRIOBP is implicated in schizophrenia

**Figure 1:**
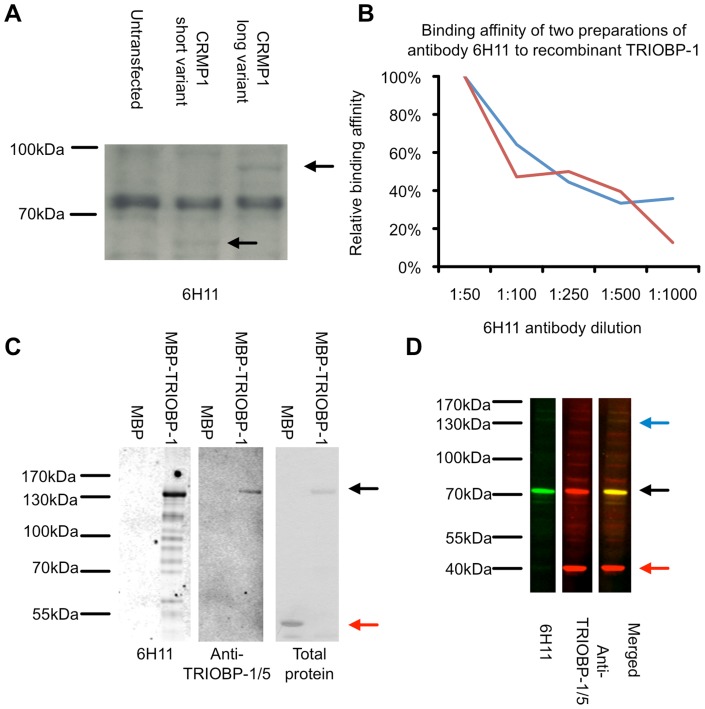
Antibody 6H11 detects TRIOBP-1 as a major epitope. (**A**) While the 6H11 antibody is able to detect both the long and short variants of CRMP1 when over-expressed in NLF neuroblastoma cells (black arrows), it also shows strong affinity to an additional 70 kDa species. (**B**) Binding strength of the schizophrenia aggregome-specific antibody 6H11 at differing dilutions to recombinant TRIOBP-1 on a protein array. Two separate preparations of the antibody from a hybridoma cell line are shown. (**C**) 6H11 recognises recombinant TRIOBP-1 protein fused to MBP (black arrow) but not recombinant MBP alone (red arrow). Some breakdown products are also visible. (**D**) Using Western blot secondary antibodies which emit at two distinct wavelengths, it can be seen that the major 70 kDa species detected by antibody 6H11 (green) coincides exactly with the major band detected by a polyclonal antibody against the C-terminus of TRIOBP-1/5 (red, 70 kDa band labelled with a black arrow). 6H11 does not recognise a 40 kDa TRIOBP species (red arrow). 6H11 thus recognises TRIOBP-1, most likely at an epitope within the N-terminal half of the protein.

We have previously described a paradigm by which a mouse is immunised with the total pooled aggregated protein of BA23 brain samples from 15 schizophrenia patients and the subsequently derived monoclonal antibodies screened for those which can specifically recognise this pool of aggregated protein from patients over an equivalent pool derived from controls, finding one such antibody [7]. Using a large dot blot array it was determined that the antibody derived from this process, named 6H11, recognised CRMP1, as was confirmed using independent antibodies raised against this protein [7], however it could also be seen to detect at least one addition protein of approximately 70 kDa in size (figure 1A). In order to systematically identify the major epitope for this antibody, two samples of it were generated from hybridoma cultures and tested on second generation protein microarray chips under GLP conditions, allowing its interactions with a range of human recombinant proteins to be probed. Potential hits were further screened on arrays containing serial dilutions of promising epitopes ranging from 20 to 0.02 fmol of protein per well. Only one protein was reliably detected in tests using both 6H11 preparations with a dilution in binding affinity that corresponded well with decreasing protein levels on the array, namely the TRIOBP-1 splice variant of TRIOBP (table S1 and figure 1B). It was confirmed that antibody 6H11 was able to recognise recombinant human TRIOBP-1 fused to Maltose Binding Protein (MBP), but not MBP alone (figure 1C) and also detected the same major 70 kDa band as a polyclonal antibody against TRIOBP in neuroblastoma cells (figure 1D). Therefore this antibody which discriminates the aggregated protein of brains with schizophrenia from similar control proteins detects TRIOBP-1 as its major epitope, implicating aggregation of TRIOBP-1 in psychiatric illness.

### The TRIOBP-1 splice variant encodes an aggregation-prone protein within the cell, in contrast to the TRIOBP-4 variant

**Figure 2:**
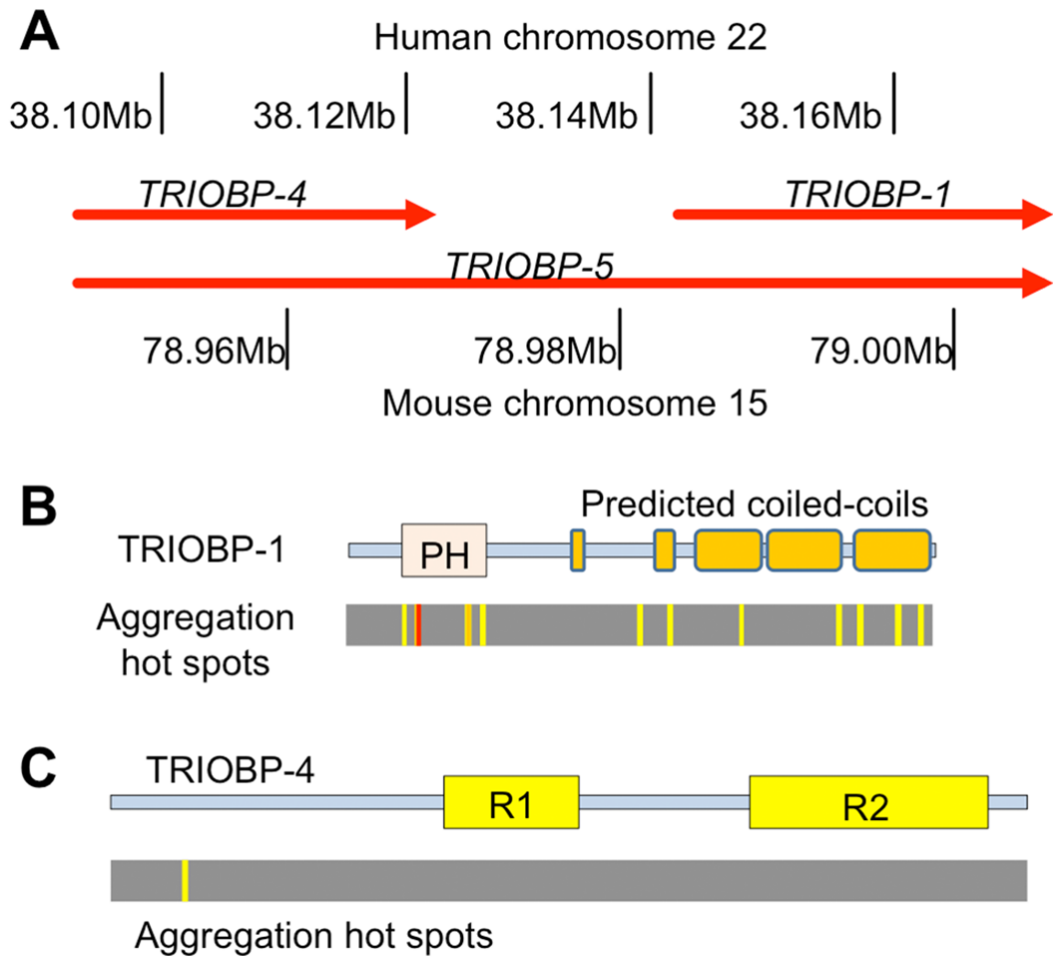
TRIOBP splice variants and their potential to form aggregates. (**A**) Relative positions of the major splice variants of TRIOBP, using the mouse nomenclature. Approximate chromosomal positions of the transcripts on human chromosome 22 and mouse chromosome 15 are indicated. (**B**) Schematic of the predicted structure of the TRIOBP-1 protein, with putative Pleckstrin homology (PH) domain and predicted coiled-coils indicated. Below are shown predicted “hot spots”, with high potential for forming protein aggregates. These were identified through analysis with six aggregation prediction paradigms from four independent servers. Hot spots were defined as stretches of 5 or more consecutive amino acids each of which was predicted to be aggregated by 3 (shown in yellow), 4 (orange) or 5 (red) of these 6 methods. (**C**) Equivalent schematic of TRIOBP-4, with two previously described repeat motifs indicated [11]. The protein is predicted to have an entirely disordered structure.

Given that the *TRIOBP* splice variants encode for an array of proteins (figure 2A), some of which share no exons with each other [9], [10], we asked which forms were capable of giving rise to aggregated protein. Initially the amino acid sequence of the principal 3′ splice variant TRIOBP-1, detected in the epitope screen, was analysed using a variety of programs which predict aggregation and/or amyloid propensity by different methodologies. Both human and mouse TRIOBP-1 were seen to contain a consistent set of regions with a high aggregation propensity (defined as a stretch of five or more amino acids each predicted to be disordered by at least half of the programs used), some of which also survived higher stringency definitions (figure 2B). By contrast, upon analysis of TRIOBP-4, the major 5′ splice variant in mouse which shares no amino acid sequence with TRIOBP-1, only a single putative aggregation region was discovered (figure 2C).

**Figure 3:**
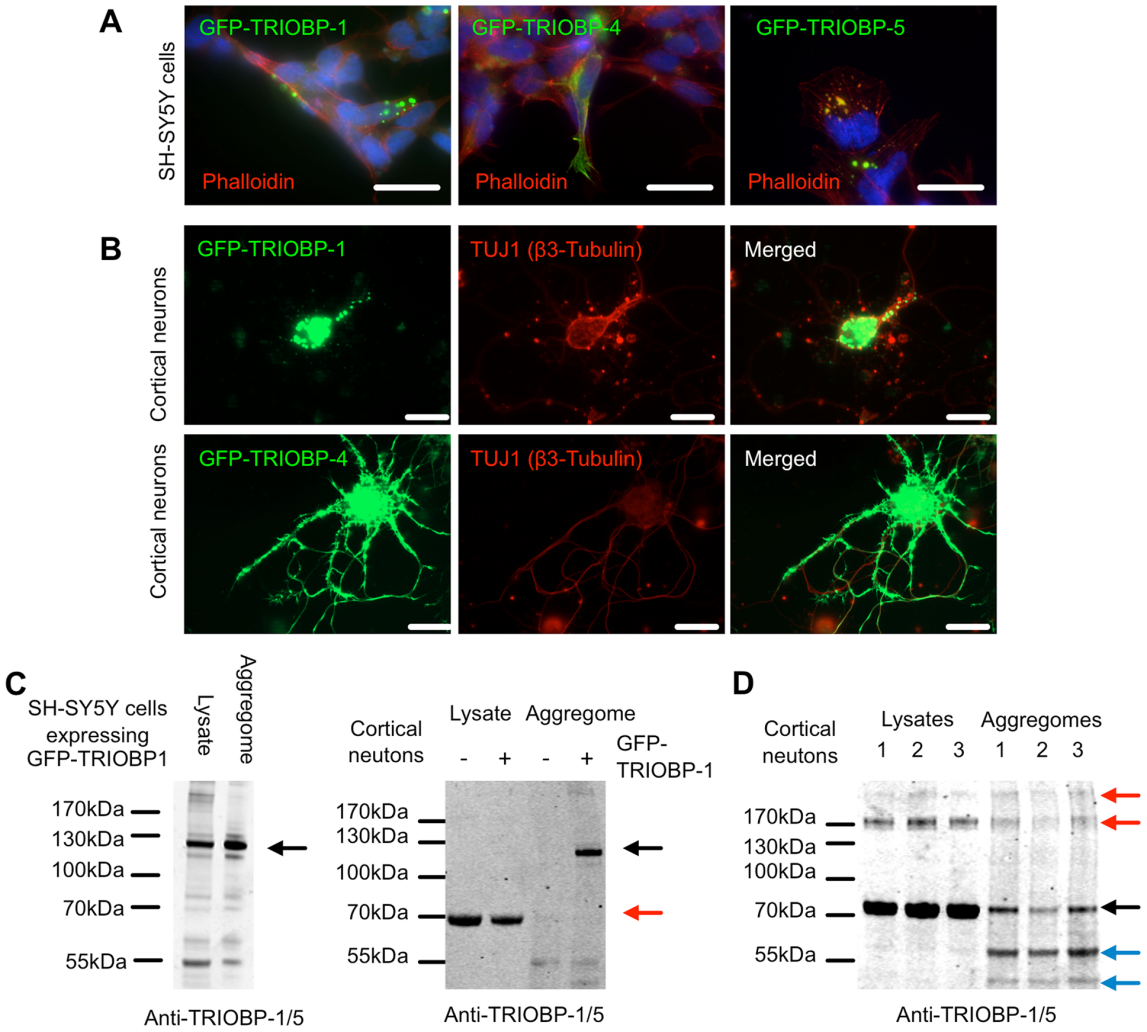
The TRIOBP-1 splice variant forms aggregates, while TRIOBP-4 does not. (**A**) GFP-fused TRIOBP-1 and TRIOBP-5 form aggregates when over-expressed in SH-SY5Y, while GFP-TRIOBP-4 does not. GFP shown in green, actin cytoskeleton revealed by fluorescent phalloidin is shown in red, DAPI-stained nuclei shown in blue. Scale bars: 20 µm. (**B**) Similarly, GFP-TRIOBP1 forms aggregates when over-expressed in rat cortical neurons (harvested at embryonic day 18, transfected at 13 days *in vitro*, fixed after 14 days *in vitro*), while TRIOBP-4 does not. GFP shown in green, neuron specific β3-tubulin antibody TUJ1 shown in red. Scale bars: 20 µm. (**C**) Upon transfection into SH-SY5Y (left panel) or rat primary cortical neurons (transfected after 13 days *in vitro* and lysed 24 hours later, right panel), over-expressed GFP-TRIOBP-1, labelled with black arrows, is seen by Western blot to be in the purified aggregated fraction. Endogenous TRIOBP can also be seen, particularly in the cortical neuron blot in which the transfection was less effective (red arrow). (**D**) Three sets of rat cortical neurons were lysed at 21 days *in vitro* and their aggregomes purified revealing the presence of TRIOBP-1 (black arrow), long variants such as TRIOBP-5 (red arrows) and shorter splice variants of the *TRIOBP* 3′ region (blue arrows) to be consistently present in this insoluble fraction. Based on the antibody used, such shorter variants would be predicted to be those which share amino acid sequence with the C-terminal half of TRIOBP-1. In all Western blots, aggregomes are enriched 10-fold relative to lysates.

Next, these predictions were tested in the cell by transfecting SH-SY5Y neuroblastoma cells with TRIOBP-1, TRIOBP-4 or TRIOBP-5 (a long variant spanning the whole locus and encompassing the TRIOBP-1 and TRIOBP-4 reading frames), each fused to GFP for detection. Consistently, both TRIOBP-1 and TRIOBP-5 formed puncta of aggregated protein upon over-expression, while TRIOBP-4 was instead seen to associate with the actin cytoskeleton, as determined by co-localisation with phalloidin (figure 3A) and fitting with that which would be expected of the endogenous protein [11]. Similarly when over-expressed in rat primary neuron cultures, GFP-TRIOBP-1 was seen to exist mainly in large aggregates in the cell body and, to a lesser extent, in neurites (figure 3B). In contrast GFP-TRIOBP-4 showed a more ubiquitous expression pattern, with particularly strong staining along the neurite periphery (figure 3B), presumably indicative of over-activity of TRIOBP-4-related actin bundling [11]. This was consistent with protein microarray screening having detected a segment of TRIOBP-1 as being the epitope recognised by the schizophrenia aggregome antibody 6H11 and with the bioinformatics analysis. This GFP-fused TRIOBP-1 protein could also be found in the purified aggregomes of both SH-SY5Y neuroblastoma cells and rat primary cortical neurons 24 hours after they had been transfected with the corresponding vector (figure 3C). Shorter, as yet uncharacterised endogenous TRIOBP-1 species were also seen to co-aggregate with the over-expressed protein.

Finally, to confirm that the endogenous TRIOBP-1 protein could also form aggregates, rat primary cortical neurons were grown for 21 days *in vitro* and then lysed. Upon purification of their insoluble aggregome fraction and probing with an anti-TRIOBP1/5 antibody, species representative of TRIOBP-1, long variants such as TRIOBP-5 and shorter C-terminal splice variants were seen (figure 3D). Thus, it appears that spontaneous protein aggregation is a feature of TRIOBP proteins encoded for by the 3′ end of the locus. Given that the principal TRIOBP species detected was 70 kDa, consistent with TRIOBP-1, and that TRIOBP-1 was the protein detected by the initial protein array screen, we decided to focus on its aggregation propensity over that of TRIOBP-5 at this time.

### TRIOBP aggregation occurs in non-mitotic cells and through coiled-coil interaction

**Figure 4:**
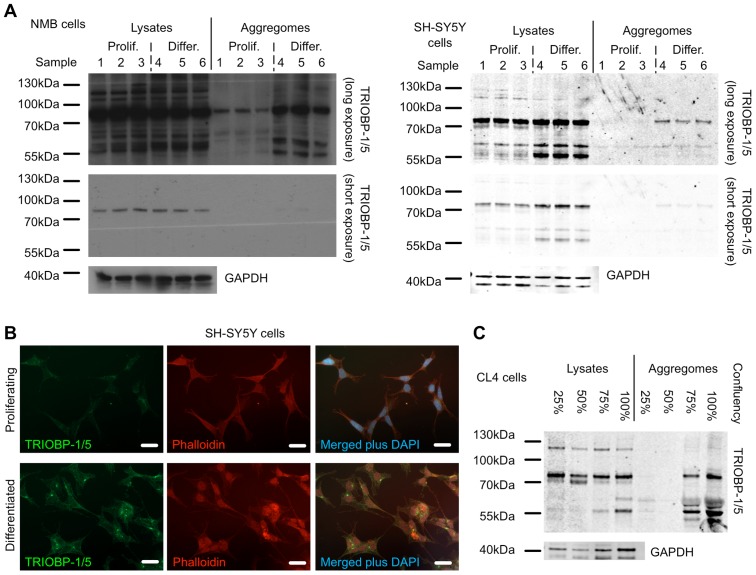
Increased aggregation of TRIOBP-1 in post-mitotic cell culture. (**A**) Western blots of total lysates and purified aggregome fractions from SH-SY5Y and NMB cells which have been harvested during proliferation (“Prolif.”, samples 1–3) or following six days differentiation (“Diff.”, samples 4–6), along with their corresponding insoluble aggregome fractions. Antibody staining reveals endogenous TRIOBP-1 to be present to a greater extent in the aggregome fraction in differentiated, non-mitotic cells. In order to allow clear viewing of both the TRIOBP-1 in the lysates and aggregomes, the same image is shown at two different lengths of exposure. GADPH is shown as a loading control. (**B**) An antibody which detects both TRIOBP-1 and TRIOBP-5 (green) demonstrates the presence of bright punctate endogenous TRIOBP structures in SH-SY5Y cells which have been differentiated, but not in cells which are still proliferating, consistent with an increase in TRIOBP-1 aggregation in these cells. Images were taken using the same microscope settings for direct comparison. Actin is visualised by phalloidin in red and DAPI in blue, scale bars: 20 µm. (**C**) Western blot of CL4 epithelial cell lysates and aggregomes taken from cell culture layers of varying confluency (approximate percentage of surface area covered with cells indicated). TRIOBP staining shows that endogenous TRIOBP-1 as well as shorter splice variants have a much higher aggregation propensity as the cells become confluent and mitosis becomes rarer.

Given that neurons are non-mitotic, it can be hypothesised that any propensity of TRIOBP-1 to aggregate would lead to accumulation of such insoluble aggregates over time, in the absence of regular cycles of cell division to dilute them out. To test this, advantage was taken of the fact that NMB and SH-SY5Y neuroblastoma cells both express endogenous TRIOBP-1 protein, to a high level in the case of NMB, and that they can each be induced to differentiate into neuron-like morphologies by treatment with dopamine or retinoic acid/PMA respectively [26], [27]. In these cells low levels of endogenous TRIOBP could consistently be detected in purified aggregome fractions using an antibody raised against the C-terminal region of TRIOBP-1 (figure 4A), demonstrating its natural tendency to aggregate. This was seen for a 70 kDa species, representing the TRIOBP-1 isoform, along with shorter 55–60 kDa species, which most likely represent as yet undescribed shorter C-terminal splice variants or proteolytically processed forms of TRIOBP. (Shorter 3′ splice variants overlapping with *TRIOBP-1* can be seen on the UCSC Genome Browser [42], Human February 2009 assembly, although these would be predicted to have sizes of only around 30 kDa). Furthermore, upon differentiation, the levels of aggregated TRIOBP-1 were seen to be raised in the NMB cells and to a lesser extent in the SH-SY5Y (figure 4A). When SH-SY5Y cells treated in this way were examined by immunofluorescent staining (figure 4B), it could be seen that bright points of endogenous TRIOBP-1 or 5 appeared in the differentiated cells which were not apparent in the proliferating cells. Together these findings are consistent with naturally aggregating TRIOBP accumulating in post-mitotic cells such as neurons. TRIOBP is also known to play a role in the cadherin-based adherens junctions of epithelial cells [12]. TRIOBP was therefore also examined in cultured CL4 epithelial cells and it was found that, while there was little evidence of TRIOBP-1 aggregates during proliferation, it became abundant in cells in which proliferation was inhibited by confluency of the cell layer in the culture dish (figure 4C), demonstrating that TRIOBP-1 aggregation is not specific to neuron-like cells.

**Figure 5:**
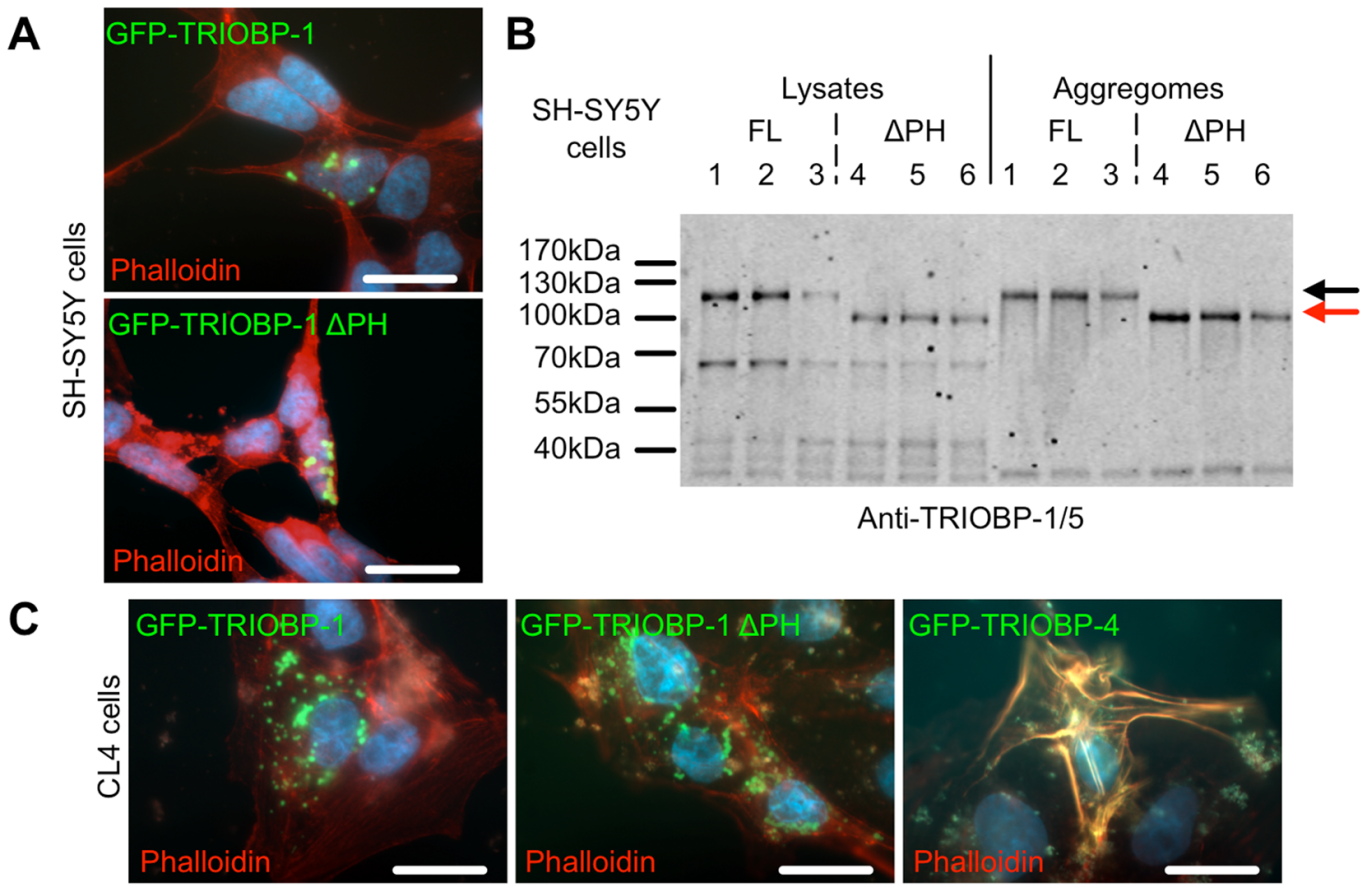
Aggregation of TRIOBP-1 does not occur via its Pleckstrin homology domain. (**A**) GST-TRIOBP-1 lacking its Pleckstrin homology domain (ΔPH) forms aggregates when expressed in SH-SY5Y neuroblastoma cells in the same manner as the full length construct. GFP shown in green, actin cytoskeleton revealed by fluorescent phalloidin (red), DAPI-stained nuclei shown in blue. (**B**) Western blot of three SH-SY5Y lysates transfected with full length (FL, samples 1–3, shown with a black arrow) or ΔPH GFP-TRIOBP-1 (samples 4–6, shown with a red arrow) along with their purified aggregome fraction, reveals no apparent difference in aggregation of TRIOBP-1 following removal of its Pleckstrin homology domain. Endogenous TRIOBP protein is also visible. (**C**) In CL4 epithelial cells, both full length and ΔPH GFP-TRIOBP-1 form insoluble aggregates while GFP-TRIOBP4 does not. GFP shown in green, actin cytoskeleton revealed by fluorescent phalloidin (red), DAPI-stained nuclei shown in blue. In all Western blots, aggregomes are enriched 10-fold relative to the lysates. In all microscopy images, scale bars: 20 µm.

Bioinformatics analysis suggested two potential mechanisms for TRIOBP-1 aggregation (figures 2B & 2C) either through α-helix-based aggregation of its coiled-coil C-terminus or through β-sheet-based aggregation of its predicted Pleckstrin homology domain. To test this latter hypothesis, a version of the GFP-TRIOBP-1 construct with this domain deleted (GFP-TRIOBP-1ΔPH) was generated and the effect of its expression in SH-SY5Y construct versus the full length construct tested. No difference in aggregation propensity was detected through either visualisation of the GFP signal by microscopy (figure 5A) or through Western blotting of purified cell aggregomes (figure 5B). Similarly, both the full length TRIOBP-1 and TRIOBP-1ΔPH aggregated when over-expressed in CL4 epithelial cells, while the TRIOBP-4 construct did not (figure 5C). It therefore appears that the TRIOBP-1 protein aggregates through one or more of its coiled-coil domains. This is consistent with the detection of 55–60 kDa species of aggregated TRIOBP in figures 3D, 4A and 4B using an antibody raised against the C-terminal region of TRIOBP-1, implying that these forms of aggregation-prone endogenous TRIOBP would contain the C-terminal coiled coil regions of TRIOBP-1 but not necessarily its N-terminal PH domain.

### Over-expression of TRIOBP-1 affects the morphology of Neuroscreen-1 cells

**Figure 6:**
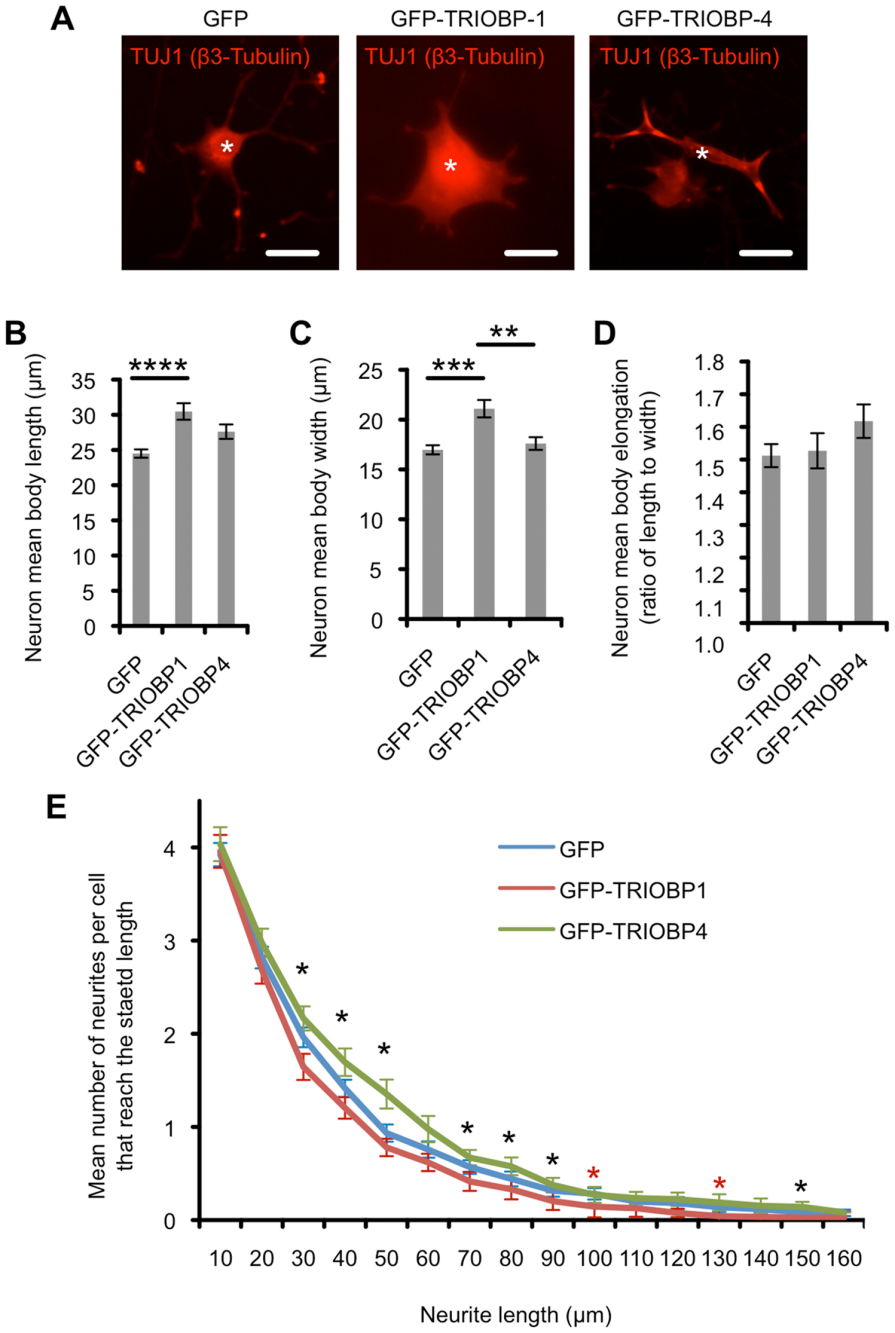
The effect of TRIOBP expression on Neuroscreen-1 cells. (**A**) Examples of NS-1 cells transfected with GFP alone (n = 181), GFP-TRIOBP-1 (n = 86) or GFP-TRIOBP-4 (n = 86). Transfected cells are indicated by white asterisks. Total cell body is visualised in red using the TUJ1 antibody, scale bars: 20 µm. (**B**) NS-1 cells transfected with GFP-TRIOBP1 show significantly longer cell bodies than those expressing GFP alone. Expression of GFP-TRIOBP-4 causes a more modest increase in length compared to GFP alone. (**C**) NS-1 cells transfected with GFP-TRIOBP1 show significantly wider cell bodies than those expressing GFP alone. (**D**) There is no significant difference in the degree of cell body elongation of NS-1 cells transfected with either GFP alone, GFP-TRIOBP1 or GFP-TRIOBP4. (**E**) Sholl analysis of NS-1 neurite growth following transfection with GFP, GFP-TRIOBP-1 or GFP-TRIOBP-4. The mean number of neurites per cell reaching a range of distances from the cell body is displayed for each transfection type. Only the first 160nm are shown as less than 5% of cells displayed neurites longer than this. Longest neurite recorded was 280 nm. Black asterisks show lengths at which the expressed protein type has a significant effect on neurite number by the Kruskal-Wallis one-way analysis of variance, while red asterisks indicate that in addition GFP-TRIOBP-1 has a significant effect over GFP by the Mann-Whitney U test, after correction for multiple testing. In all graphs, *: p<0.05, **: p<0.01, ***: p<0.001, ****: p<0.0001.

In order to determine whether the presence of TRIOBP-1 aggregates may have an adverse effect on neuronal development, Neuroscreen-1 cells, a subclone of the PC-12 rat neuron-like cell line, were transfected with constructs encoding either GFP, GFP-TRIOBP-1 or GFP-TRIOBP-4. Cells were then differentiated with nerve growth factor for four days before being fixed and visualised with the TUJ1 anti-β-tubulin antibody. Images were taken of transfected cells (GFP: n = 181, GFP-TRIOBP-1: n = 118, GFP-TRIOBP-4: n = 85, examples in figure 6A) and the images analysed for neuronal morphology as well as the dimensions of the cell body in a blinded manner.

Cells expressing the aggregating TRIOBP-1 construct were seen to have significantly larger cell bodies than those expressing GFP alone, with the mean length of the longest axis of the cell body being approximately 25% greater for GFP-TRIOBP-1 transfected cells (p = 6.1×10^−5^, figure 6B). There was also a significant increase in the width of the cell body, defined as the longest axis perpendicular to the length (p = 2.6×10^−4^, figure 6C), with no difference in the degree of elongation of the cell (the ratio of the length and width, figure 6D), thus TRIOBP-1 aggregates lead to an increase in total cell body volume. Expression of non-aggregating GFP-TRIOBP-4 instead also led to a more subtle increase in cell body length relative to those expressing GFP alone, however this fell just short of significance after Bonferroni correction (p = 0.058, figure 6B). There was also a subtle decrease in neurite length following GFP-TRIOBP-1 transfection, compared to GFP alone. Intriguingly, transfection of non-aggregating GFP-TRIOBP-4 led to an increase in neurite outgrowth length, but not total number of neurite protrusions, suggesting that TRIOBP-4, or else the equivalent section of long TRIOBP variants such as TRIOBP-5, may have a proactive role in neurodevelopment in addition to its known roles in the ear.

## Discussion

Following similar demonstrations for DISC1, dysbindin and CRMP1 previously, we here present data to indicate that TRIOBP-1 is a protein which forms aggregates specifically in at least a subset of brains of patients with chronic mental illness. The importance of identifying such specifically aggregating proteins is twofold. Firstly, this is through providing insight into the cellular mechanisms of the development of psychiatric illness. That specific proteins aggregate in psychiatric illness indicates at a minimum that the processes by which they are expressed, folded and/or degraded are disrupted in illness, however the potential for these aggregates to have active detrimental effects within neurons has also been demonstrated in principal through the ability of aggregated DISC1 to induce aggregation of other proteins [6], [7] and to interfere with cellular functions [43]. Secondly, while protein aggregation in the brain in itself provides a valid biomarker, but one that is not practical for diagnosis, it is plausible that aggregation in the brain would be concomitant with proteostasis disruption in other more accessible tissues.

It is notable that the previously described proteins which fulfil these criteria of aggregation in psychiatric illness were all encoded for by genes which have independently been associated with these conditions. Indeed, the aggregation propensity of both DISC1 and dysbindin was initially investigated specifically based on these genetic associations [5], [6], while CRMP1 aggregation was discovered a hypothesis-free manner [7] but nevertheless identified a protein encoded for by a chromosomal region previously implicated in psychiatric illness [44], [45]. In contrast, any putative role for TRIOBP-1 in psychiatric illness is novel, and therefore the findings presented here have the potential to be the first instance of aggregation-based proteomics implicating a novel protein in their pathology.

In terms of aggregation mechanism, TRIOBP-1 appears to have a natural tendency to form aggregates, a propensity which is enhanced in non-mitotic cells such as neurons in which cell division cannot provide a mechanism of diluting the accumulated protein. Structurally, it is most likely that TRIOBP-1 forms aggregates through large-scale interaction of the coiled-coil domains of different TRIOBP-1 molecules, in analogy to the presumed mechanism of aggregation of DISC1 [46]. The alternative, aggregation occurring through the interaction of β-strands, is seemingly discounted as deletion of the only region of the TRIOBP-1 protein with a predicted β-strand, the Pleckstrin homology domain, has no effect on the aggregation propensity of over-expressed protein, while under the same circumstances, TRIOBP-4 shows no such propensity.

Notably, the fact that the proteins derived from the *TRIOBP* gene are established to modulate the actin cytoskeleton [9], [11] implies that disruption of these proteins may have an effect on the developmental morphology of cells including neurons. As an initial test of this theory, we have demonstrated that expression of TRIOBP-1 aggregates in the neuron-like NS-1 cell line leads to aberrantly large cell bodies, with a suggestion that they may also inhibit neuronal outgrowth. The potential therefore exists that naturally occurring TRIOBP-1 aggregates may directly affect neuronal development and/or function.

During the course of this work, we have also examined the *TRIOBP-4* splice variant found at the 5′ end of the locus, discovering that its expression in a neuron-like cell line led to an increase in neurite outgrowth. Previous RT-PCR expression experiments have suggested that TRIOBP-4 is not expressed in the brain, at least in mouse, being instead predominantly found in the retina and inner ear [10]. Transcripts encoding long variants such as TRIOBP-5 were however detected in mouse brain cDNA [10], and it is therefore feasible that such long variants expressed in the brain and encompassing the TRIOBP-4 sequence may play a role in neurodevelopment. The possibility of TRIOBP-4 being expressed in the brain at earlier developmental stages in mouse or showing difference expression in other mammals also cannot be discounted.

Finally, the fact that mutations in the *TRIOBP* locus are associated with deafness ([10], [15]–[17]) is interesting given that there exists data supporting a degree of comorbidity between hearing loss and psychiatric illness. Amongst a cohort of over 50,000 Swedish conscripts, incidence of schizophrenia was increased (odds ratio, OR = 1.81) in those with severe hearing loss [47], while in the World Health Organisation world health survey of over 220,000 individuals of diverse ethnicity, hearing problems were more common in individuals with psychotic symptoms, both with (OR = 2.27) and without (OR = 1.56) a diagnosis of psychosis [48]. One recent case control study in the Swedish population also showed that hearing impairment at age 4 was associated with increased risk of developing non-affective psychosis in later life (OR = 6.0) [49], while a survey of Flemish general practise data examined the reverse scenario, but found that the presence of a diagnosis of psychosis had no significant association with the subsequent development of hearing or vision problems [50]. While it seems unlikely that one single missense polymorphism in the *TRIOBP* locus could account for both hearing loss and psychiatric symptoms within a single patient, given that the former have to date all been found in the 5′ splice variants, while the aggregating TRIOBP-1 protein is encoded for by a 3′ variant, it is possible nevertheless that defects in either expression of the gene or that a co-inherited combination of polymorphisms within the *TRIOBP* locus may be a cause of comorbidity of these conditions in some patients. It is also possible that long splice variants, such as *TRIOBP-5*, which span the entire locus may play roles in both sets of conditions. It is also noticeable that in one family from Pakistan in which three siblings displayed both schizophrenia and hearing impairment, along with epilepsy, homozygosity mapping implicated the causative mutation(s) to lie in one of two chromosomal regions, one of which includes the *TRIOBP* locus [51].

Identification of proteins specifically aggregating in the brains of subsets of patients with major mental illness provides a strategy for identification of proteins important in the development of these conditions independent from, but complementary to, tradition and high-throughput genetic analyses. To our existing trio of DISC1, dysbindin and CRMP1, we hereby present TRIOBP-1 as a fourth protein with the potential to meet these criteria and have extensively characterised its aggregation propensity, as well as demonstrating their effect on the morphology of a neuron-like cell line. Further experiments in material from patients are now required in order to investigate how general this accumulation of TRIOBP-1 aggregation in the brain is and thus its degree of clinical relevance to the pathology of chronic mental illness.

## Supporting Information

Table S1 **Affinity of two preparations (A and B) of the 6H11 antibody to a subset of recombinant proteins, selected based on an initial protein microarray.** Only one protein, TRIOBP-1 (highlighted), detects signal from both preparations and shows a general decrease in antibody binding with reducing levels of protein used.
(XLSX)
